# Exercise interventions to improve bone mineral density in athletes participating in low-impact sports: a scoping review

**DOI:** 10.1186/s12891-025-08316-5

**Published:** 2025-01-20

**Authors:** Anders Gulbrandsen Florvåg, Øyvind Angelshaug Berg, Ola Drange Røksund, Gøril Tvedten Jorem, Bård Erik Bogen

**Affiliations:** 1 Faculty of Health and Social Sciences, Western Norway University of Applied Sciences, Bergen, Norway; 2 Department of Pediatric and Adolescent Medicine, Haukeland University Hospital, Bergen, Norway; 3 Research support. Bergen, Western Norway University of Applied Sciences, Bergen, Norway; 4 Department of Global Public Health and Primary Care, University of Bergen, Bergen, Norway

**Keywords:** Bone density, Cycling, Exercise, Osteopenia, Osteoporosis, Swimming

## Abstract

**Background:**

Athletes participating in low-impact sports such as cycling and swimming are at increased risk for low bone mineral density, which may lead to long-term health issues. Exercise is known to increase bone mineral density, but there is little knowledge of the effects of this in athletes participating in low-impact sports. This review aims to identify potential exercise interventions that could improve bone health in these athletes.

**Methods:**

There appears to be little research on this topic, and we addressed the research question using a scoping review to get a broad overview of the research literature. The scoping review was conducted following the methodological framework of Arksey and O´Malley. A literature search was conducted May 2024 in SPORTDiscus, Web of Science, Scopus, MEDLINE, EMBASE, Cinahl, Cochrane, and Google Scholar. The Consensus on Exercise Reporting Template was used to evaluate the reporting of the exercise intervention(s).

**Results:**

A total of 2528 studies were screened and assessed for eligibility. Five studies met the inclusion criteria reporting results of exercise interventions on bone mineral density in cyclists and swimmers. Different designs were applied, and study populations varied. Five populations were explored; one cohort of adolescent swimmers (both sexes), one cohort of female Olympic artistic swimmers, one cohort of elite road cyclists (both sexes), one cohort of male competitive amateur cyclists, and one cohort of trained to well-trained cyclists (both sexes).

**Conclusion:**

This scoping review found that resistance training, plyometric exercises, whole-body vibration, and a combined jumping exercise with collagen supplementation show promising results in improving bone mineral density in athletes participating in cycling and swimming. However, there is limited evidence due to few identified studies, with varying study designs and inconsistent exercise reporting, highlighting the need of further research to better understand how different training approaches can improve bone health in these athletes.

**Supplementary Information:**

The online version contains supplementary material available at 10.1186/s12891-025-08316-5.

## Background

Bone mass and bone strength increase from childhood to adulthood, and then decreases in old age, with an accelerated bone loss for postmenopausal women [1]. Generally, low bone strength is not noticed until a fracture occurs [2]. Crucially, in accordance with Wolff´s law, bone strength develops with mechanical loading of the skeleton, as mechanotransduction transforms these mechanical forces into cellular signals that promote structural adaptations in the bone [3, 4]. Frost´s mechanostat theory suggests that bones undergo resorption when exposed to mechanical loading below a certain threshold, leading to loss of bone mass [5]. Therefore, even highly active athletes may be prone to bone loss due to low impact in their sports [3]. Acute fractures lead to long recovery times and significant financial costs for individual athletes and teams, prompting injury prevention efforts and the optimization of management [6].

Engaging in exercise and physical activity is associated with positive effects on bone health and can help in prevention of osteoporosis and osteopenia [7, 8]. Consequently, athletes, especially those involved in high-impact sports, tend to have higher bone mineral density (BMD) than non-athletes [9, 10]. Conversely, participation in low-impact sports is associated with risk of low BMD [11–14] and may not improve bone health [10]. Thus, when comparing the BMD of cyclists and swimmers to both non-athlete controls and athletes in high-impact sports, cyclists and swimmers consistently show lower BMD [15].

A systematic review from 2016 reports reduced BMD in the hip, lumbar spine, leg, and pelvis in cyclists, and reduced BMD in the lower limbs in swimmers, when compared to controls [11]. Andersen et al. [16] found that 10 of 19 Norwegian elite endurance cyclists had low BMD (Z-score ≤ − 1), and one had osteoporosis (Z-score ≤ − 2). Medelli et al. [17] found that two-thirds of a professional team of road cyclists had abnormally low BMD. Bellver et al. [18] found that BMD values of female Olympic swimmers and Olympic artistic swimmers were comparable to those of sedentary controls. Rowing, similar to cycling and swimming, is considered a low impact or non-weight bearing sport, as the legs are not in contact with the ground or support body weight [19]. However, elite rowers do not have lower BMD compared to controls [20, 21]. This may be due to the fact that rowers are exposed to greater compression loads, particularly on the trunk, compared to the mechanical loads typically experienced in cycling and swimming [21].

Peak bone mass (PBM) is typically achieved by the third decade of life [10]. Attaining a high PBM early in life can predict higher bone mass and a lower risk of osteoporosis or osteopenia later in life [22]. The age at which peak athletic performance is achieved varies across different sports and genders, typically falling between 20 and 30 years old [23]. Therefore, intense competition and training in low-impact sports during the period of PBM attainment could potentially have adverse effects on the long-term bone health of these athletes. Individuals with osteoporosis and low BMD are advised to participate in weight-bearing exercise and resistance training to improve bone health [24]. However, it remains unclear whether similar interventions yield comparable effects in athletes participating in low-impact sports.

Thus, the aim of this scoping review is to provide an overview of potential exercise interventions that could improve BMD in athletes participating in low-impact sports.

## Methods

### Design

This scoping review was conducted following the five-stage methodological framework of Arksey and O´Malley [25]. The Preferred Reporting Items for Systematic Reviews and Meta-Analyses extension for Scoping Reviews (PRISMA-ScR) checklist was used to guide the review process [26]. Additionally, the Joanna Briggs Institute (JBI) scoping review manual [27] and the PRISMA-ScR checklist were applied to develop the scoping review protocol [26]. The protocol was published on the Open Science Framework on July 31, 2024, and can be accessed at 10.17605/OSF.IO/Y2DZH.

### Identifying the research question

The Population, Intervention and Outcome (PIO) framework, adapted from the Population, Intervention, Comparison and Outcome (PICO) [28], was used to define our research question (Table 1) and develop the search strategy. Our research question is *“What research exists regarding exercise interventions for improving bone mineral density in athletes participating in cycling or swimming?”*, as cyclist and swimmers consistently show lower BMD compared to controls [15].

**Table 1:** The population, intervention, outcome (PIO) model used to conduct the search

Population	Intervention	Outcome
Athletes participating in cycling or swimming	Exercise intervention(s)	Bone Mineral Density

### Identifying relevant studies

The planning of search terminology, selection of databases, and literature search were conducted in collaboration with GTJ, a university librarian at Western Norway University of Applied Sciences. The literature search was carried out in SPORTDiscus (EBSCO), Web of Science, Scopus, MEDLINE (Ovid), EMBASE (Ovid), Cinahl (EBSCO) and Cochrane databases. Additionally, citation, author, and reference searches of the included studies were conducted using Google Scholar. The literature search took place on May 13, 2024. The detailed search strategy and results can be found in Supplementary Material 1.

### Study selection and identification of articles

The inclusion criteria for the studies were that they must report on the effects of exercise interventions on BMD in athletes participating in cycling or swimming. Journal articles of all types of study designs published between 2000 and 2024 were included.

Studies were excluded if they involved non-athletes, female athletes with menstrual disorders, athletes with relative energy deficiency in sports (RED-S), animals or not in English, Norwegian, Swedish or Danish. Rowers were excluded as they do not have lower BMD than controls [20, 21]. Studies focusing on supplement or nutritional interventions without accompanying exercise interventions, were not included.

**Fig. 1:**
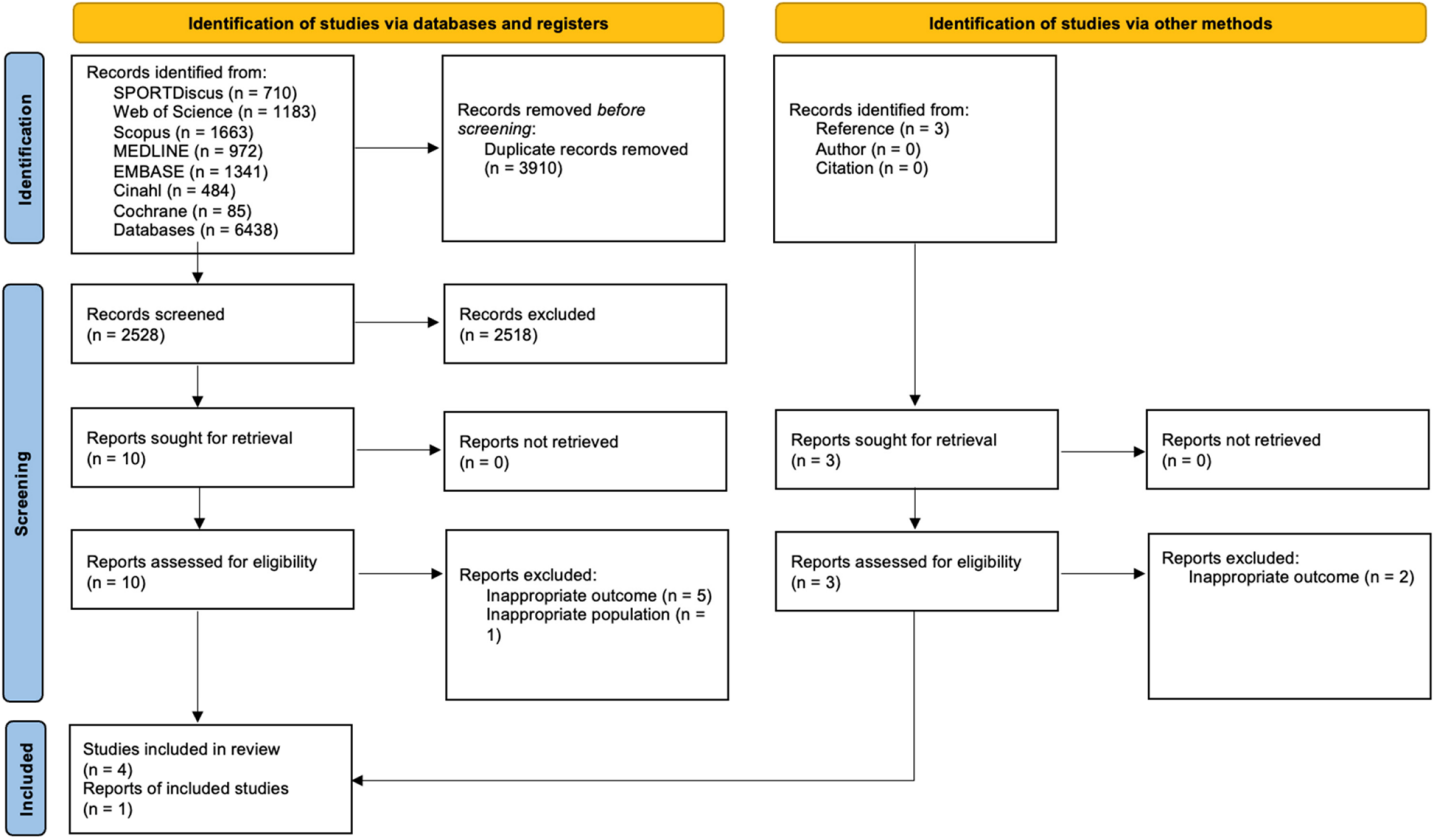
PRISMA 2020 flow diagram for new systematic reviews which included searches of databases, registers and other sources. From: Page MJ, McKenzie JE, Bossuyt PM, Boutron I, Hoffmann TC, Mulrow CD, et al. The PRISMA 2020 statement: an updated guideline for reporting systematic reviews. BMJ 2021;372:n71. 10.1136/bmj.n71. For more information, visit: http://www.prisma-statement.org/

Following the literature search, all identified articles were uploaded to EndNote, and duplicates were excluded. Since not all duplicates were identified by EndNote, the remaining articles were then uploaded to Rayyan for further screening. Titles and abstracts were independently screened by AGF and ØAB to assess eligibility based on the predetermined criteria. Full texts of potentially relevant studies were retrieved and assessed in detail individually. Reasons for exclusion of studies after reading full text were recorded and reported (Fig. 1 and Supplementary Material 2).

Disagreements between reviewers at any stage of the selection process were resolved through discussion or by involving an additional reviewer (BB/ODR). The results of the search and the study inclusion process are presented in the PRISMA flow diagram (Fig. 1).

### Data extraction and synthesis

Authors AGF and ØAB individually reviewed the included articles. Based on Arksey and O’Malley’s framework [25], the following information was extracted into a table (Table 2 and Supplementary Material  3): [1] author(s), year of publication, study location; [2] method, design, aim; [3] study population; [4] intervention(s); [5] results; [6] conclusion. The following description of exercise intervention(s) was extracted: type of intervention(s), duration in weeks and per session, frequency, and intensity, alongside the effects of the intervention on BMD in cyclists or swimmers. If the study involved a sport or activity other than cycling or swimming, the results related to BMD for these athletes were not extracted. Disagreements were resolved through discussion, or with the assistance of an additional reviewer (BB/ODR). The studies characteristics and findings were described and presented narratively in a synthesis, in accordance with Arksey and O’Malley [25].

**Table 2:**
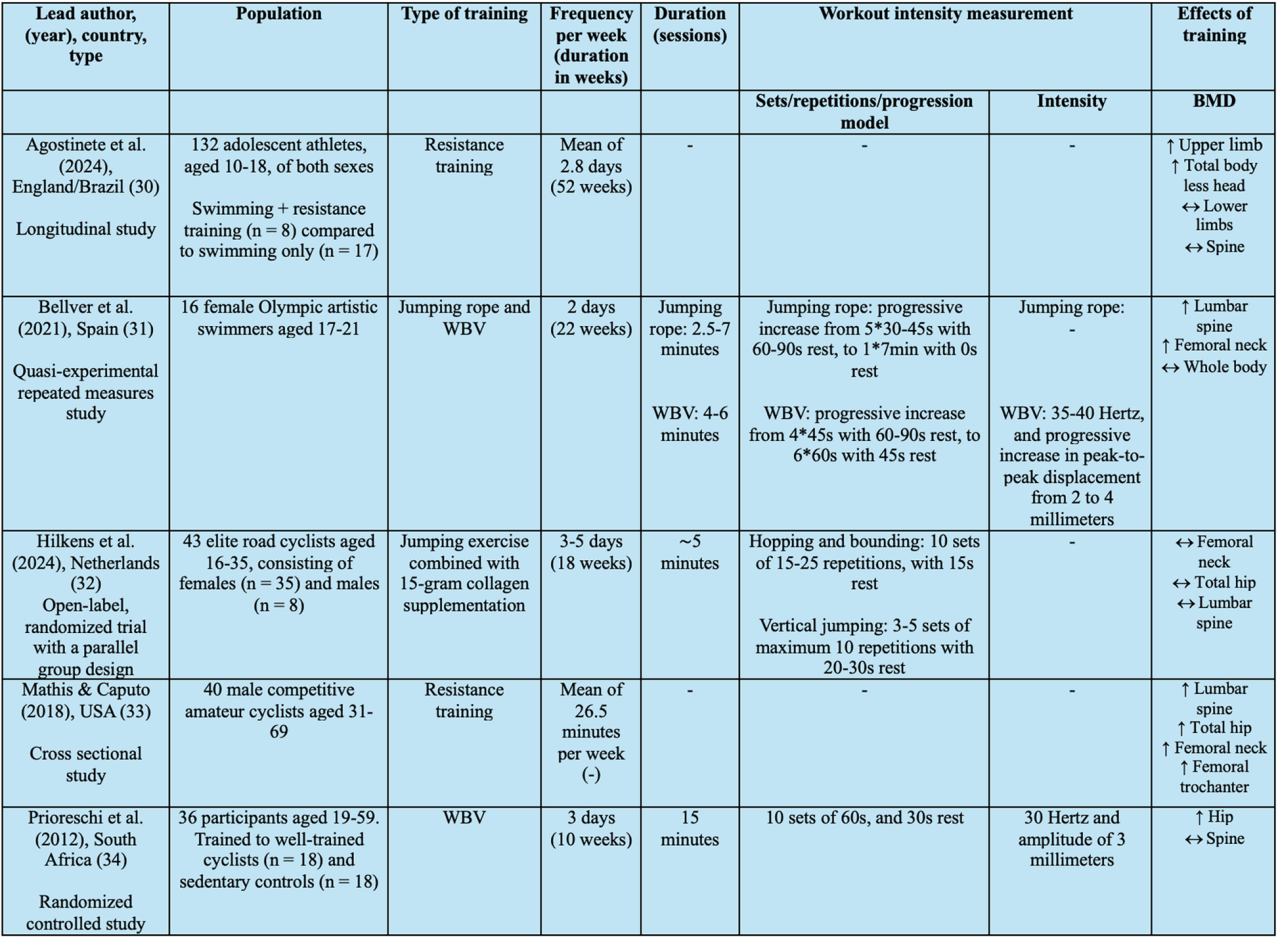
Lead author/year/country/type, population, type of training, frequency per week, duration, workout intensity measurement, effects of training

↑ = statistically significant improvement (*p* < 0.05), « = not statistically significant improvement (*p* > 0.05), - = not reported, *BMD *Bone mineral density, *s *seconds, *WBV *Whole body vibration. See Supplementary Material 3 for more detailed description of the included studies

The Consensus on Exercise Reporting Template (CERT) was used to evaluate the reporting of the exercise intervention(s) (Table 3). 16 items were assessed and scored, with a maximum obtainable score of 19. These 16 items is the minimum data set required to report exercise interventions [29]. 1 point were given if information is provided, and 0 points, if information is not sufficiently reported. AGF and ØAB independently evaluated the reporting of the interventions in the included studies. Any disagreements were resolved through discussion, or with the assistance of an additional reviewer (BB/ODR).

**Table 3:** Results of using the consensus on exercise reporting template (CERT)

		Bellver et al. 2021 [31]	Hilkens et al. 2024 [32]	Prioreschi et al. 2012 [34]	Section total
Section/topic	#				
**WHAT: materials**	**1. exercise equipment**	1	1	1	**3**
**WHO: provider**	**2. instructor(s) qualifications**	0	0	0	**0**
**HOW: delivery**	**3. performed individual or in group**	0	0	0	**0**
	**4. supervised**	1	0	0	**1**
	**5. adherence**	0	1	1	**2**
	**6. motivation strategies**	0	0	0	**0**
	**7a. rule(s) for progression**	1	1	0	**2**
	**7b. description of exercise(s) progression**	1	1	0	**2**
	**8. descriptions of exercise(s)**	1	1	1	**3**
	**9. description of home program component**	0	1	0	**1**
	**10. description of non-exercise component**	0	1	0	**1**
	**11. adverse events**	1	1	1	**3**
**WHERE: location**	**12. setting**	1	1	1	**3**
**WHEN**,** HOW**,** MUCH: dosage**	**13. detailed description of exercise intervention(s)**	1	1	1	**3**
**TAILORING: what**,** how**	**14a. generic or tailored exercise(s)**	1	1	1	**3**
	**14b. detailed description of how exercise(s) are tailored to the individual**	0	0	0	**0**
	**15. starting level**	0	0	0	**0**
**HOW WELL: planned**,** actual**	**16a. assessment of adherence or fidelity**	0	1	1	**2**
	**16b. reporting of adherence or fidelity**	1	1	0	**2**
**Total**:		**10**	**13**	**8**	

### Quality appraisal

The quality of the included studies was not assessed as this is a scoping review [25, 27].

## Results

The results regarding selection and characteristics of sources of evidence are presented. This is followed by the results of individual sources of evidence, presented in Table 2. Subsequently, a narrative synthesis, in accordance with Arksey and O´Malley [25], is presented.

### Sources of evidence

The selection process of studies is presented in a PRISMA flow diagram in Fig. 1.

Following the literature search, 2528 records were identified after removing duplicates. Four studies met the inclusion criteria [30–33]. After screening references, authors, and citations of these four included studies, one additional study was included [34]. Studies excluded after reviewing full text are presented in  Supplementary Material 2.

### Characteristics of sources of evidence

The five included studies were published between 2012 and 2024. One study was published 2024 in BMC (BioMed Central) Pediatrics [30], one was published 2021 in Journal of Bone and Mineral Metabolism [31], one was published 2024 in International Journal of Sport Nutrition and Exercise Metabolism [32], one was published 2018 in Journal of strength and condition research [33], and one was published 2012 in International journal of sports medicine [34].

### Results of individual sources of evidence

Agostinete et al. [30] conducted a 12-month longitudinal study on 91 adolescent swimmers aged 10–18, investigating the combined effects of sports participation and resistance training on areal BMD accrual. Engagement in resistance training were assessed through a face-to-face interview, and swimmers were divided into two groups: one engaging in both swimming and resistance training, and the other participating in swimming only. BMD was assessed using Dual X-ray absorptiometry (DXA) at baseline and after 12-months. The swimming and resistance training group showed significant increases in upper limb and total body without head BMD compared to the swimming only group, though changes in lower limb and spine BMD were similar between the groups. The authors suggest that professionals responsible for adolescent swimmers could implement resistance training to promote bone health [30].

Bellver et al. [31] conducted a quasi-experimental repeated measures study, across two seasons on 16 female Olympic artistic swimmers aged 17–21. They investigated the effects of a 22-week program combining jumping rope and whole-body vibration (WBV) on BMD. DXA scans conducted before and after the intervention revealed significant increases in lumbar spine and femoral neck BMD, but no significant change in whole-body BMD. The interventions were performed twice a week, and compliance was reported at 100% with minimal adverse effects (one case of tibia periostitis). The authors suggest that these findings may encourage elite artistic swimmers’ coaches to implement jumping rope and WBV in their training programs to improve BMD in female Olympic artistic swimmers [31].

Hilkens et al. [32] conducted an open-label, randomized trial with a parallel group design, with 43 elite road cyclists aged 16–35. They assessed the impact of 18-week intervention of frequent short bouts of jumping exercise combined with 15 g of dietary collagen on BMD. BMD was assessed using DXA at baseline and after the investigation, revealing preservation of femoral neck BMD in the intervention group, while there was a decrease in the control group. There was a trend towards preservation of total hip BMD, though not statistically significant. No significant changes were observed in lumbar spine or whole-body BMD. The intervention was performed five times per week, and compliance averaged 77%. One dropout was potentially related to the jumping intervention, as the participant sustained a ligament injury of the foot. The authors conclude that this combined approach could counteract the negative effects of professional cycling on bone health [32].

Mathis & Caputo [33] conducted a cross-sectional study on 40 male competitive amateur cyclists aged 31–69. Cyclists completed a self-report questionnaire that included questions about weekly minutes of weight training, and BMD was assessed using DXA. They found a strong positive correlation between weekly minutes of weight training and BMD at the lumbar spine, total hip, femoral neck, and femoral trochanter. The authors conclude that health care professionals and coaches working with male competitive cyclists are encouraged to prescribe a weight-training regimen to protect the bone health of these athletes [33].

Prioreschi et al. [34] conducted a randomized controlled trial with 18 trained to well-trained cyclists aged 19–59 to examine the effects of a 10-week WBV intervention. BMD was assessed using DXA before and after the intervention. They found a significant increase in hip BMD in the intervention group compared to controls. A significant loss in spine BMD was observed in the control group compared to the intervention group, but the difference was not significant after adjusting for baseline BMD values. WBV was performed three times a week. Average compliance was not reported. One cyclist reported back pain during the intervention, though it remains unclear if this was due to the intervention or a pre-existing condition. The authors suggest that 10-weeks may not be sufficient to fully reveal the benefits of WBV [34].

Data identified in the five individual studies that relates to the research question are presented in Table 2.

### Synthesis of results

Five studies reported various exercise interventions and their effects on BMD in cyclists and swimmers, including: resistance training, jumping rope, WBV, and a combination of jumping exercises with collagen supplementation. The studies varied in design, methods and population (Table 2), but highlighted key findings.

Resistance training has shown promising results across two different studies investigating the impact on BMD in swimmers and cyclists. Both Agostinete et al. [30] and Mathis & Caputo [33] found positive impacts of resistance training on BMD. Agostinete et al. [30] found significant increases in BMD in upper limbs and total body less head among adolescent swimmers, while Mathis & Caputo [33] found a strong positive correlation between weekly duration of resistance training and BMD at the lumbar spine, total hip, femoral neck, and femoral trochanter in male competitive amateur cyclists. However, none of the studies provided detailed descriptions of the exercises used, their duration, frequency, or intensity involved in the resistance training (Table 2).

In addition to resistance training, jumping rope and WBV have been explored as effective interventions in swimmers and cyclists. Bellver et al. [31] demonstrated that a combined jumping rope and WBV program significantly improved lumbar spine and femoral BMD in female Olympic artistic swimmers. Prioreschi et al. [34] found that WBV increased hip BMD and maintained spine BMD in trained to well-trained road cyclists. The studies had clear and structured training protocols but utilized different approaches to facilitate progression. Bellver et al. [31] employed a progressive approach, whereas Prioreschi et al. [34] did not incorporate progression in their training intervention (Table 2).

A combined approach of jumping exercises and collagen supplementation has also showed promising results. Hilkens et al. [32] found that frequent short bouts of jumping exercise combined with supplementation of 15 gram of collagen helped preserve femoral neck BMD in elite road cyclists. The intervention program was both structured and progressive, with variations introduced every other week to optimize bone adaptation (Table 2).

### Results of exercise reporting

The studies of Agostinete et al. [30] and Mathis & Caputo [33] were not evaluated due to their non-experimental study designs. The studies of Bellver et al. [31], Hilkens et al. [32] and Prioreschi et al. [34] were evaluated, as they described protocols for their interventions. See Table 3 for scoring.

Total score for CERT varied from 8 to 13 points (of 19). Two studies obtained a score of more than 50%, with a score of 10 and 13 [31, 32]. Item 2 (instructor(s) qualifications), 3 (performed individual or in group), 6 (motivation strategies), 14b (detailed description of how exercise(s) are tailored to the individual) and 15 (starting level) were not sufficiently reported by the three studies.

## Discussion

This scoping review included five studies that reported on the effects of exercise interventions on BMD in cyclists and swimmers.

The duration of interventions in Bellver et al. [31], Hilkens et al. [32] and Prioreschi et al. [34] varied between 22, 18 and 10 weeks, respectively. The bone remodeling process generally require approximately 17 weeks for cortical bone and 29 weeks for trabecular bone [35]. Despite these average remodeling times, the studies reported positive changes in BMD even with relatively short intervention periods (Table 2). Although all the studies had interventions durations shorter than the average remodeling period for trabecular bone, they still observed positive changes in BMD at both the lumbar spine [31] and femoral neck [31, 32, 34], which are primarily trabecular bone regions [36]. This suggest that shorter periods of jumping rope, WBV, and a combined jumping exercise and collagen supplementation leads to positive bone outcomes. However, it remains unclear whether longer duration of the interventions would further improve BMD in cyclists and swimmers.

Agostinete et al. [30] and Mathis & Caputo [33] were the only studies that investigated the effects of resistance training on BMD. However, due to the non-experimental designs of these studies, specifics of the resistance training were not well documented (Table 2). Without this information, it is challenging to give specific recommendations regarding resistance training for cyclists and swimmers. In contrast, the details of jumping rope, WBV, and jumping exercise combined with collagen supplementation are well documented. These interventions come with detailed protocols, making them reproducible and easier to implement in training routines.

Although it is uncertain whether the increase in BMD achieved during an intervention period is sustained afterward, there is evidence that regular weight-bearing activity throughout the year has beneficial effects on BMD [24]. This is crucial as both male and female cyclists have been observed to experience a yearly loss of 1–2% in hip BMD during training and competition [37]. Given the high prevalence of low BMD in retired elite cyclists, it is possible that BMD values may not fully recover after their professional cycling careers [38]. Additionally, high-volume cycling and swimming does not contribute to increased BMD [11]. These observations highlights the needs for implementing exercise interventions to counteract the negative effects of cycling and swimming on BMD.

To ensure compliance among athletes and coaches, it can be beneficial to tailor interventions to the specific demands of their respective sports. Specificity in training emphasizes that the more closely training exercises resemble the demands and movements of the sport, the better the effect of the training will be transferred to actual performance [39]. Both amateur [14] and elite cyclists [40] express concerns about the potential negative impact of resistance training on their performance. Additionally, amateur cyclists have been reported to be unwilling to include plyometric training [14]. Despite these concerns and perceptions, resistance training can enhance cycling performance [41], as well as swimming performance [42]. Similarly, plyometric exercises have been shown to improve performance in both cycling [43] and swimming [44]. WBV has been found to improve anaerobic [45] and sprint performance [46] in well-trained cyclists, although it does not appear to benefit performance in swimmers [47].

Given these findings, resistance training and plyometric exercise emerge as particularly relevant strategies for improving both BMD and performance in cyclists and swimmers. However, it is important to note that the details regarding resistance training in Agostinete et al. [30] and Mathis & Caputo [33] are not well documented (Table 2). Consequently, it is challenging to make specific recommendations regarding resistance training. Bellver et al. [31], Hilkens et al. [32] and Prioreschi et al. [34] scored 10, 13 and 8 points respectively, according to the CERT (Table 3), making it difficult to draw conclusions due to insufficient exercise reporting.

Additionally, insufficient reporting of exercise loading parameters, such as intensity, frequency and duration, are critical for understanding the osteogenic effects of the exercise interventions described in Table 2 [3, 5]. Mechanical loads can also vary widely due to individual factors such as muscle mass, with greater muscle mass placing more stress on bone tissue and contributing to increased bone mass [48]. The limited reporting constrains the practical application of these findings and highlights the need for future studies to standardize and quantify loading parameters to enable more precise exercise recommendations.

### Limitations

Due to the dearth of studies on this topic, we decided not to do a traditional systematic review or a meta-analysis. Scoping reviews are meant for an overview of a topic and allow for the inclusion of different study designs. However, scoping reviews may be unsuited for firm conclusions based on research evidence.

Our search identified only five studies, which constitutes a small body of evidence and limits the strengths and generalizability of our findings. The studies included in this scoping review showed considerable variations in important variables including age, gender, athletic ability, and particular sports, making it difficult to draw consistent conclusions across a range of demographics. Additionally, variability in study designs and methodologies, as summarized in Table 2, poses challenges in comparing results meaningfully. As seen in Table 3, the lack of consistency in exercise reporting adds to discrepancies that complicate the synthesis of the data. Furthermore, dietary factors, which were not addressed in this scoping review, are also likely to have a significant impact on bone health [49] and may influence the effectiveness of the interventions. Further studies should aim to address these limitations.

## Conclusion

In conclusion, there is notable limitations in the existing literature particularly regarding the quantification and standardization of loading protocols. There is limited evidence due to few identified studies, with varying study designs and inconsistent exercise reporting. The identification of only five studies highlights a clear gap in research regarding the impact of exercise interventions on BMD in these athletes. However, resistance training, plyometric exercises, WBV, and a combined jumping exercise with collagen supplementation show promising results in improving BMD in athletes participating in cycling and swimming, particularly in areas such as the spine and hip. Further research is needed to better understand how different training approaches can improve bone health in cyclists and swimmers.

## Supplementary Information

Supplementary Material 1.

Supplementary Material 2.

Supplementary Material 3.

## Data Availability

No datasets were generated or analysed during the current study.

## Abbreviations

BMC: BioMed Central
BMD: Bone mineral density
CERT: Consensus on exercise reporting template
DXA: Dual x-ray absorptiometry
JBI: Joanna Briggs Institute
PBM: Peak bone mass
PICO: Population, intervention, comparison and outcome
PIO: Population, intervention and outcome
PRISMA: Preferred Reporting Items for Systematic Reviews and Meta-Analyses
PRISMA-ScR: Preferred Reporting Items for Systematic Reviews and Meta-Analyses extension for Scoping Reviews
RED-S: Relative energy deficiency in sports
WBV: Whole-body vibration
