# Supplementary material for: Exercise interventions to improve bone mineral density in athletes participating in low-impact sports: a scoping review
Source: BMC Musculoskelet Disord. 2025 Jan 20;26:73. doi: 10.1186/s12891-025-08316-5 (PMC11744971; doi:10.1186/s12891-025-08316-5)
Supplement: Supplementary file 1 — Supplementary Material 1. [file 12891_2025_8316_MOESM1_ESM.docx]

Additional file 1. Documentation of literature search.

Documentation of literature search.

| Librarian | Name: Gøril Tvedten Jorem  Workplace: Western Norway University of Applied Sciences, Research support. Bergen, Norway.  Email: goril.tvedten.jorem@hvl.no |
| --- | --- |

# SPORTDiscus

Date for search: May 13, 2024

Number of hits: 710 (before deduplication)

Comments:

Documentation of search:

| # | Query | Results |
| --- | --- | --- |
| S1 | DE "ATHLETES" | 57,572 |
| S2 | TI athlete* OR AB athlete* OR KW athlete* | 178,743 |
| S3 | S1 OR S2 | 191,130 |
| S4 | DE "BONE density" | 4,590 |
| S5 | TI bone N2 densit* OR AB bone N2 densit* OR KW bone N2 densit* | 6,317 |
| S6 | TI BMD OR AB BMD OR KW BMD | 2,787 |
| S7 | S4 OR S5 OR S6 | 7,377 |
| S8 | DE "EXERCISE" | 100,464 |
| S9 | DE "EXERCISE therapy" | 8,649 |
| S10 | DE "SPORTS" | 128,912 |
| S11 | DE "PHYSICAL activity" | 32,269 |
| S12 | DE "SWIMMING" OR DE "CYCLING" OR DE "ROWING" | 67,948 |
| S13 | TI ( exercis* or training or sport* ) OR AB ( exercis* or training or sport* ) OR KW ( exercis* or training or sport* ) | 780,897 |
| S14 | TI ( (motor or physical or locomotor) N0 activit* ) OR AB ( (motor or physical or locomotor) N0 activit* ) OR KW ( (motor or physical or locomotor) N0 activit* ) | 61,111 |
| S15 | TI ( physical N0 (condition* OR fitness OR endurance) ) OR AB ( physical N0 (condition* OR fitness OR endurance) ) OR KW ( physical N0 (condition* OR fitness OR endurance) ) | 66,279 |
| S16 | TI ( bicycle OR cycle OR bicycling OR cycling OR rowing OR swim* ) OR AB ( bicycle OR cycle OR bicycling OR cycling OR rowing OR swim* ) OR KW ( bicycle OR cycle OR bicycling OR cycling OR rowing OR swim* ) | 146,316 |
| S17 | S8 OR S9 OR S10 OR S11 OR S12 OR S13 OR S14 OR S15 OR S16 | 953,849 |
| S18 | S3 AND S7 AND S17 | 710 |

# Web of Science

Date for search: May 13, 2024

Number of hits: 1183 (before deduplication)

Comments:

Documentation of search:


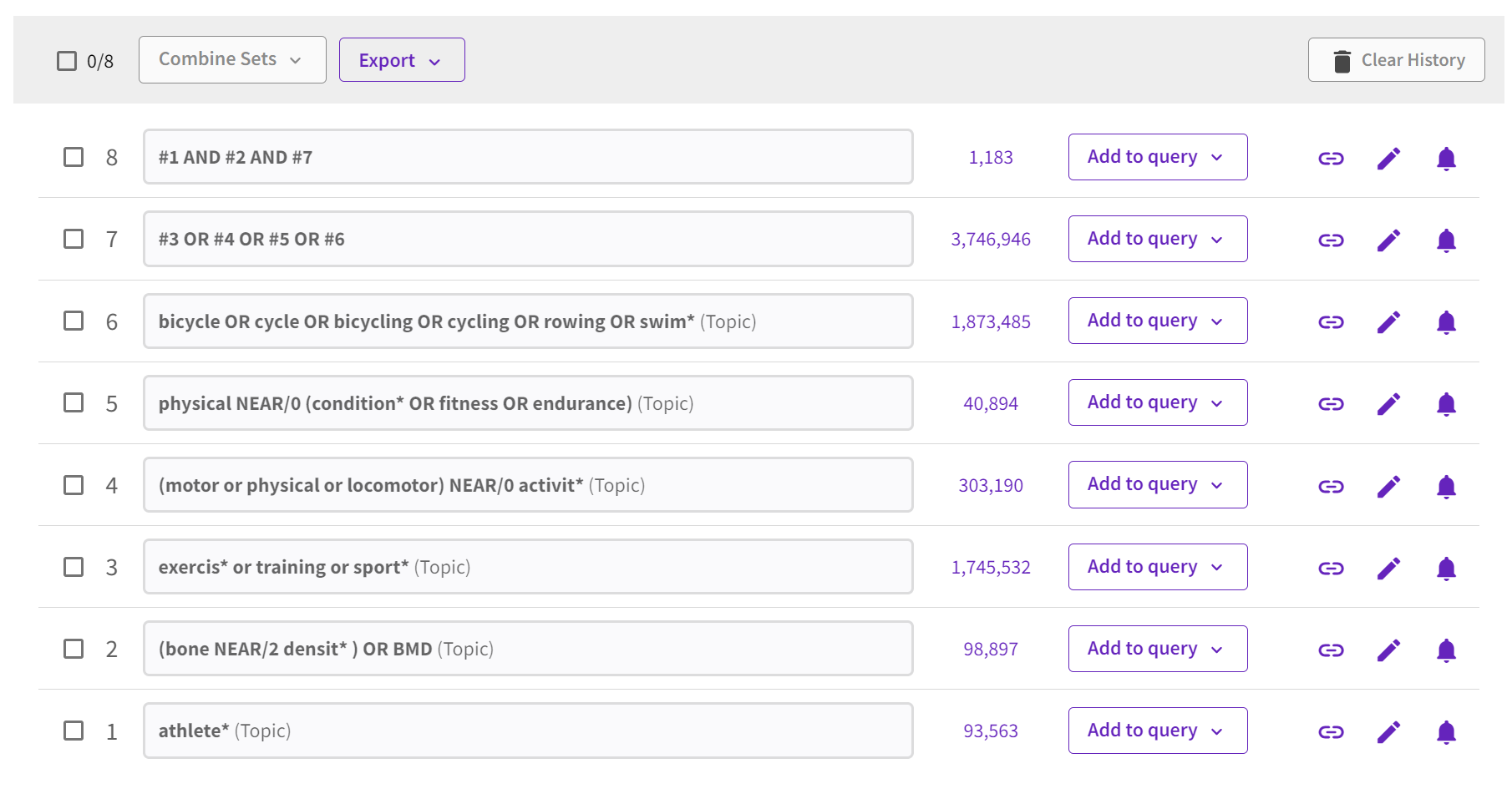


# Scopus

Date for search: May 13, 2024

Number of hits: 1663 (before deduplication)

Comments:

Documentation of search:


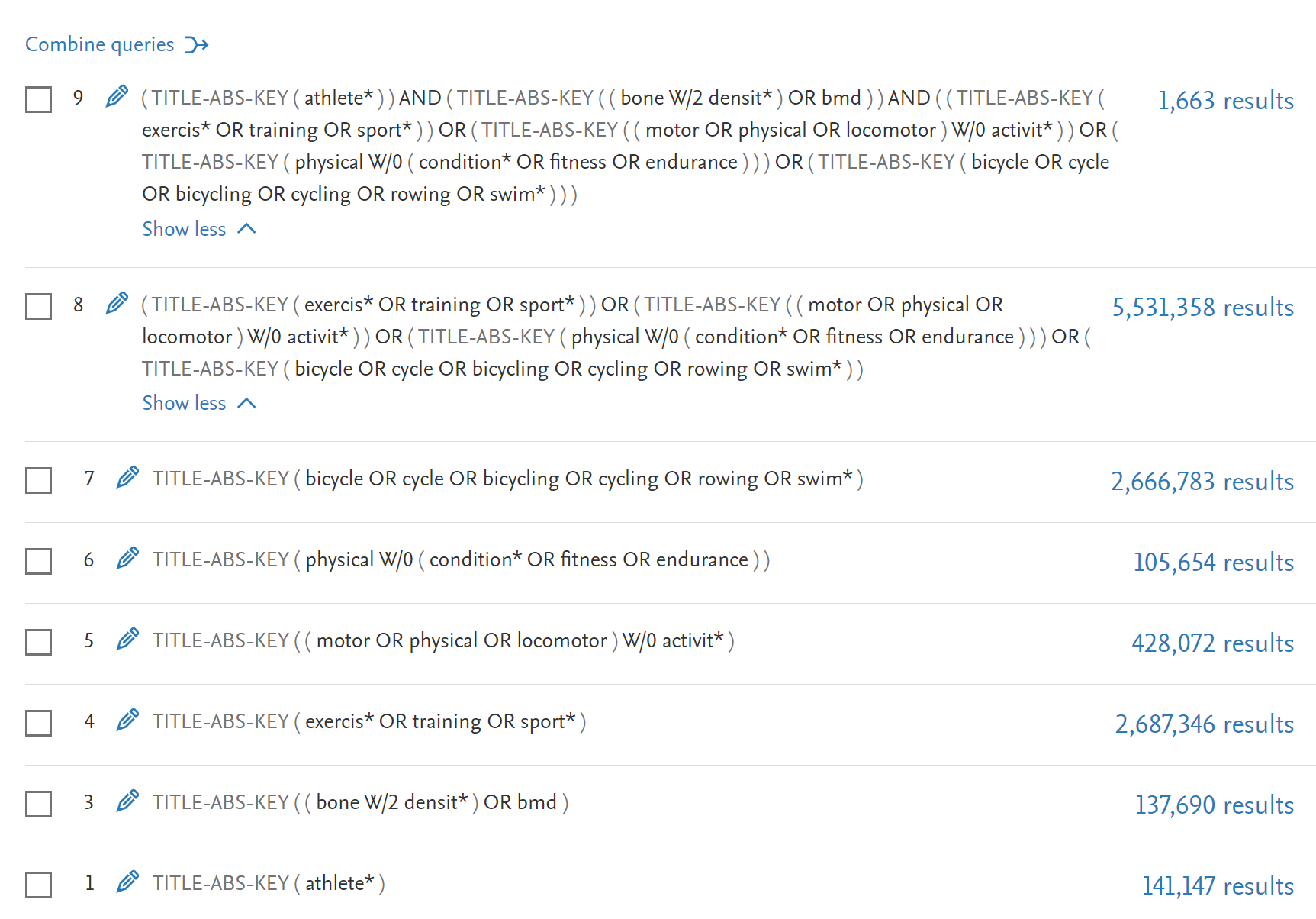


# Medline

Date for search: May 13, 2024

Number of hits: 972 (before deduplication)

Comments:

Documentation of search:

Database: Ovid MEDLINE(R) ALL <1946 to May 10, 2024>
Search Strategy:
1  exp Athletes/ (21919)
2  athlete*.ti,ab. (70255)
3  1 or 2 (74217)
4  Bone Density/ (62287)
5  (bone adj2 densit*).ti,ab. (65528)
6  BMD.ti,ab. (35943)
7  4 or 5 or 6 (90093)
8  exp Exercise/ (256449)
9  exp Exercise Therapy/ (65931)
10  sports/ or bicycling/ or swimming/ (67852)
11  (exercis* or training or sport*).ti,ab. (959009)
12  ((motor or physical or locomotor) adj activit*).ti,ab. (197920)
13  (physical adj (condition* or fitness or endurance)).ti,ab. (21970)
14  (bicycle or cycle or bicycling or cycling or rowing or swim*).ti,ab. (700551)
15  8 or 9 or 10 or 11 or 12 or 13 or 14 (1849796)
16  3 and 7 and 15 (972)

# Embase

Date for search: May 13, 2024

Number of hits: 1341 (before deduplication)

Comments:

Documentation of search:

Database: Embase <1974 to 2024 Week 19>
Search Strategy:
1  athlete/ (64701)
2  athlete*.ti,ab. (83523)
3  1 or 2 (100302)
4  bone density/ (118313)
5  (bone adj2 densit*).ti,ab. (94923)
6  BMD.ti,ab. (60919)
7  4 or 5 or 6 (142076)
8  exercise/ (360461)
9  kinesiotherapy/ (39980)
10  sport/ or cycling/ (76019)
11  (exercis* or training or sport*).ti,ab. (1261997)
12  ((motor or physical or locomotor) adj activit*).ti,ab. (261761)
13  (physical adj (condition* or fitness or endurance)).ti,ab. (27862)
14  (bicycle or cycle or bicycling or cycling or rowing or swim*).ti,ab. (877123)
15  8 or 9 or 10 or 11 or 12 or 13 or 14 (2376823)
16  3 and 7 and 15 (1341)

# Cinahl

Date for search: May 13, 2024

Number of hits: 484 (before deduplication)

Comments:

Documentation of search:

| # | Query | Results |
| --- | --- | --- |
| S1 | (MH "Athletes") | 14,859 |
| S2 | TI athlete* OR AB athlete* | 37,332 |
| S3 | S1 OR S2 | 42,986 |
| S4 | (MH "Bone Density") | 20,388 |
| S5 | TI bone N2 densit* OR AB bone N2 densit* | 16,686 |
| S6 | TI BMD OR AB BMD | 7,823 |
| S7 | S4 OR S5 OR S6 | 26,185 |
| S8 | (MH "Exercise+") | 131,762 |
| S9 | (MH "Therapeutic Exercise+") | 65,358 |
| S10 | (MH "Sports") | 9,831 |
| S11 | (MH "Swimming") | 4,859 |
| S12 | (MH "Cycling") | 9,831 |
| S13 | (MH "Rowing") | 1,092 |
| S14 | TI ( exercis* or training or sport* ) OR AB ( exercis* or training or sport* ) | 390,759 |
| S15 | TI ( (motor or physical or locomotor) N0 activit* ) OR AB ( (motor or physical or locomotor) N0 activit* ) | 81,460 |
| S16 | TI ( physical N0 (condition* or fitness or endurance) ) OR AB ( physical N0 (condition* or fitness or endurance) ) | 8,161 |
| S17 | TI ( bicycle or cycle or bicycling or cycling or rowing or swim* ) OR AB ( bicycle or cycle or bicycling or cycling or rowing or swim* ) | 88,417 |
| S18 | S8 OR S9 OR S10 OR S11 OR S12 OR S13 OR S14 OR S15 OR S16 OR S17 | 594,618 |
| S19 | S3 AND S7 AND S18 | 484 |

# Cochrane

Date for search: May 13, 2024

Number of hits: 1 review, 84 trials (before deduplication)

Comments:

Documentation of search:

Search Name:

Date Run: 13/05/2024 19:00:31

Comment:

ID Search Hits

#1 MeSH descriptor: [Athletes] explode all trees 1740

#2 (athlete*):ti,ab,kw (Word variations have been searched) 9402

#3 #1 or #2 9402

#4 MeSH descriptor: [Bone Density] explode all trees 6022

#5 (bone NEAR/2 densit*):ti,ab,kw (Word variations have been searched) 14869

#6 (BMD):ti,ab,kw (Word variations have been searched) 7297

#7 #4 or #5 or #6 15581

#8 MeSH descriptor: [Exercise] explode all trees 38714

#9 MeSH descriptor: [Exercise Therapy] explode all trees 21703

#10 MeSH descriptor: [Sports] explode all trees 22087

#11 MeSH descriptor: [Bicycling] explode all trees 2128

#12 MeSH descriptor: [Swimming] explode all trees 652

#13 (exercis* or training or sport*):ti,ab,kw (Word variations have been searched) 250094

#14 ((motor or physical or locomotor) NEXT activit*):ti,ab,kw (Word variations have been searched) 50156

#15 (physical NEXT (condition* OR fitness OR endurance)):ti,ab,kw (Word variations have been searched) 13159

#16 (bicycle OR cycle OR bicycling OR cycling OR rowing OR swim*):ti,ab,kw (Word variations have been searched) 82507

#17 #8 OR #9 OR #10 OR #11 OR #12 OR #13 OR #14 OR #15 OR #16 338725

#18 #3 AND #7 AND #17 85
