# Supplementary material for: Exercise interventions to improve bone mineral density in athletes participating in low-impact sports: a scoping review
Source: BMC Musculoskelet Disord. 2025 Jan 20;26:73. doi: 10.1186/s12891-025-08316-5 (PMC11744971; doi:10.1186/s12891-025-08316-5)
Supplement: Supplementary file 2 — Supplementary Material 2. [file 12891_2025_8316_MOESM2_ESM.docx]

Additional file 2. Studies excluded after reviewing full text.

Identification of studies via databases and registers:

Inappropriate outcome (n = 5)

1. Beshgetoor D, Nichols JF, Rego I. Effect of Training Mode and Calcium Intake on Bone Mineral Density in Female Master Cyclists, Runners, and Non-Athletes. International journal of sport nutrition and exercise metabolism. 2000;10(3):290–301.
2. Gómez‐Bruton A, González‐Agüero A, Matute‐Llorente A, Julián C, Lozano‐Berges G, Gómez‐Cabello A, et al. Effects of Whole Body Vibration on Tibia Strength and Structure of Competitive Adolescent Swimmers: A Randomized Controlled Trial. PM & R. 2018;10(9):889–97.
3. Lee N, Kim J. A review of the effect of swim training and nutrition on bone mineral density in female athletes. Journal of exercise nutrition & biochemistry. 2015;19(4):273–9.
4. Vlachopoulos D, Barker AR, Ubago-Guisado E, Williams CA, Gracia-Marco L. The effect of a high-impact jumping intervention on bone mass, bone stiffness and fitness parameters in adolescent athletes. Archives of osteoporosis. 2018;13(1):128–128.
5. Wirth K, Keiner M, Fuhrmann S, Nimmerichter A, Haff GG. Strength Training in Swimming. International journal of environmental research and public health. 2022;19(9):5369.

Inappropriate population (n = 1)

1. Bubanj S, Mitković M, Gašić T, Mazić S, Stanković R, Radovanović D, et al. The Impact of Resistance Training Program on the Muscle Strength and Bone Density in Adolescent Athletes. Acta Facultatis Medicae Naissensis. 2018;35(3):201–15.

Identifications of studies via other methods:

Inappropriate outcome (n = 2)

1. Gómez-Bruton A, González-Agüero A, Matute-Llorente A, Julián C, Lozano-Berges G, Gómez-Cabello A, et al. Do 6 months of whole-body vibration training improve lean mass and bone mass acquisition of adolescent swimmers? Archives of osteoporosis. 2017;12(1):69–69.
2. Marin-Puyalto J, Gomez-Cabello A, Gonzalez-Aguero A, Matute-Llorente A, Gomez-Bruton A, Jürimäe J, et al. Effects of whole-body vibration training on bone density and turnover markers in adolescent swimmers. J Pediatr Endocrinol Metab. 2020 May 26;33(5):623–30.
