# Supplementary material for: Exercise interventions to improve bone mineral density in athletes participating in low-impact sports: a scoping review
Source: BMC Musculoskelet Disord. 2025 Jan 20;26:73. doi: 10.1186/s12891-025-08316-5 (PMC11744971; doi:10.1186/s12891-025-08316-5)
Supplement: Supplementary file 3 — Supplementary Material 3. [file 12891_2025_8316_MOESM3_ESM.docx]

**Additional file 3
Overview of the Included Studies by Author, Year, Country of Origin, Method/Design/Aim, Study Population, Intervention, Results, and Conclusion**

Author, year and country Method/design/aim Study population Intervention Results Conclusion

*The authors conclude that participation in resistance training improves areal bone mineral density in upper limbs and total body less head in adolescent swimmers. Exercise professionals working with adolescent swimmers could implement resistance training to improve bone health and performance, while also reducing the risk of injuries.*

*132 Brazilian adolescents, aged 10-18, of both sexes were included. After 12 months 91 adolescent athletes (males: n = 70, females: n = 21) completed the follow-up measures. Final sample consisted of impact sport group (n = 66): basketball (n = 23), tennis (n = 15), baseball (n = 10), gymnastics (n = 10) and track & field (n = 8). Non-impact group: swimming (n = 25).*

*The athletes were divided into four subgroups: impact sports only (n = 45), impact sports + resistance training (n = 21), swimming only (n = 17), and swimming + resistance training (n = 8).*

*Dropouts (n=41) were observed due to interrupting sports participation, transfer to another squad in a different city or declining to participate in the follow-up.*

*Type: Resistance training

Duration: Not reported

Frequency: Mean of 2.8 days/week (±1.5).

Intensity: Not reported

Description: The researchers assessed resistance training engagement at baseline through a face-to-face interview. Participants were first asked if they were engaged in resistance training. If yes: how long, and how many days/week.*

*Swimming + resistance training group presented higher areal bone mineral density accrual in upper limbs than swimmers only (p = 0.001, effect size: 1.70 (large)) over the 12 months period. Swimming + resistance training group presented higher areal bone mineral density accrual in total body less head compared with swimming only (p = 0.026, effect size: 1.12 (large)). In lower limbs and spine, swimming + resistance training showed similar changes in areal bone mineral density compared to swimming only group (p = 0.720, effect size 0.18 (trivial)).*

*The aim of this longitudinal study was to analyze the combined effects of sports participation and resistance training on areal bone mineral density accrual in adolescent athletes participating in swimming and impact sports.*

*Adolescents were divided into four sub-groups according to resistance training participation.*

*Bone mineral density was assessed with dual X-ray absorptiometry at baseline and follow up after 12 months.*

*The study was published and analyzed in England but conducted in Brazil.*

*Agostinete et al. 2024, England/Brazil (30)*

*Resistance training presents beneficial effects on bone development of adolescent engaged in swimming but not in impact sports: ABCD Growth Study*

**Additional file 3.** (Continued)

Author, year and country Method/design/aim Study population Intervention Results Conclusion

*Type: jumping rope and whole-body vibration training.*

*Duration: 22 weeks. Jumping rope minutes/session: 2.5-3.45 (week 1-4), 6 (week 5-13), 7 (week 14-22). Whole body vibration minutes/session: 4 (week 1-9), 6 (week 10-22).*

*Frequency: 2 times/week.*

*Intensity: Jumping rope: week 1-4: 5*30-45s work and 60-90s rest. Week 5-13: 6*60s work, and 45s rest. Week 14-22: 1*7min, with 0s breaks. Whole body vibration: week 1-9: 4*45s, 60-90s rest. Week 10-22: 6*60s, 45s rest. The training consisted of vibrations between 35 and 40 Hertz, and peak-to-peak displacement of 2 millimeter (week 1-9), and 4 millimeter (week 10-22).*

*Description: The intervention was always conducted before water technical training. The whole-body vibration training was performed with the knee joint angle in 90 °, while standing on a platform and holding the hands on the handle. The participants wore the same gymnastic shoes to standardize the damping of the vibration of the footwear.*

*A statistically significant difference was found between pre-intervention (start of second season) and post-intervention (end of second season) in lumbar spine (p = 0.002, effect size: 1.046 (large)) and femoral neck bone mineral density (p = 0.02, effect size: 0.77 (moderate)). A trend towards significance was found in whole body bone mineral density (p= 0.08, effect size: 0.65 (moderate)). The artistic swimmers gained 2% in bone mineral density over the intervention period.*

*The average jumping rope and whole-body vibration compliance was 100%. One participant reported an episode of tibia periostitis due to self-limited whole body vibration training and continued the study.*

*The authors conclude that the findings may encourage coaches of elite artistic swimmers to implement jumping rope and whole-body vibration training into their regular training programs to improve bone mineral density.*

*Bellver et al. 2021, Spain (31)*

*Jumping rope and whole-body vibration program effects on bone values in Olympic artistic swimmers*

*16 female Olympic artistic swimmers aged 17-21. The participants were an Olympic team who have trained more than ten years at a high intensity, volume and frequency.*

*A quasi-experimental repeated measures study, presenting a longitudinal analysis of two artistic swimmer´s seasons.*

*The aim of the study was to assess whether a 22-week jumping rope and whole-body vibration program, in addition to regular training, would lead to an increase in bone mineral density in a team of female Olympic artistic swimmers compared to data from the same athletes without interventions.*

*Dual X-ray absorptiometry investigation was conducted on three occasions: start of first season (2013), start of second season (2014), and after 22 weeks use of the specific training protocol.*

**Additional file 3.** (Continued)

Author, year and country Method/design/aim Study population Intervention Results Conclusion

*43 elite road cyclists aged 16-35 years, consisted of females (n = 35), males (n = 8).*

*Dropouts (n = 7). Interventions group dropout (n = 4): injuries or personal reasons. Control group dropout (n = 3): injuries (n = 2), health issues (n = 1). Final sample size (n = 36): females (n = 28), males (n = 8).*

*Hilkens et al. 2024. Netherlands (32)*

*Jumping Exercise Combined With Collagen Supplementation Preserves Bone Mineral Density in Elite Cyclists*

*Type: jumping exercise combined with 15-gram collagen supplementation.*

*Duration: 18 weeks, ∼5 min/session.*

*Frequency: 3 times/week the first two weeks, with gradual increase to 5 times/week to prevent injuries.*

*Intensity: Hopping and bounding consisted of 10 sets of 15-25 repetitions, with 15s rest between sets. Vertical jumping consisted of 3-5 sets of a maximum 10 repetitions with 20-30s of rest in between sets.*

*Description: The jumping were multidirectional hopping and vertical jumping, to optimize bone adaptation response based on loading factors. Every other week, the intervention would change the workout regimen and exercise selection to provide stimuli and increase compliance. Participants were told not to incorporate the jumping intervention with resistance training or cycling. Instead, the participants were told to separate the intervention and their regular training with a couple of hours.*

*Supplement: Before each jumping intervention the participants were instructed to ingest collagen supplement (Vita supply), consisting of 13.5-gram protein (hydrolyzed Type 1 collagen), 0-gram carbohydrates, 0-gram fat, 60 milligrams vitamin C, and 7.5 microgram vitamin D, mixed with tap water or non-caloric flavors. A researcher monitored compliance and potential adverse effects by contacting the participants weekly.*

*Of all participants, low bone mineral density was found at the hip (19%), femoral neck (25%), lumbar spine (39%) and whole body (14%).*

*Femoral neck bone mineral density decreased in the control group, while being preserved in the intervention group (Time x Treatment: p = < 0.01). A similar effect was observed for total hip bone mineral density but did not reach statistical significance (p = 0.08). No Time x Treatment interactions were observed for lumbar spine (p = 0.62) and whole body (p = 0.57) bone mineral density.*

*Femoral neck bone mineral density, effect size: 0.23 (large). Total hip bone mineral density, effect size: 0.9 (moderate). Lumbar spine bone mineral density: effect size: 0.1 (small). Whole body bone mineral density: effect size: 0.1 (small)*

*One of the dropouts was potentially related to the jumping intervention: ligament injury of the foot.*

*Compliance was 84% the first nine weeks, and 55% in the last nine weeks. The overall compliance for the 18-week intervention period was 77% (total sessions completed per week).*

*The authors conclude that frequent short sessions of jumping exercise combined with collagen supplementation, can positively affect bone mineral density of the femoral neck in elite road-race cyclists during off seasons. This approach seems promising for counteracting the negative effects of professional cycling on bone health.*

18-week open-label, randomized trial with a parallel group design was conducted during the off-season of elite-cyclists.

The aim of this study was to assess the impact of frequent short sessions of jumping exercise performed five times weekly, combined with the ingestion of 15-gram dietary collagen, on bone mineral density in male and female elite road-race cyclists.

Bone mineral density was assessed with dual X-ray absorptiometry before and after the 18-week intervention.

**Additional file 3.** (Continued)

Author, year and country Method/design/aim Study population Intervention Results Conclusion

*Mathis & Caputo, 2018, USA (33)*

*Resistance Training Is Associated With Higher Lumbar Spine and Hip Bone Mineral Density in Competitive Male Cyclists*

The authors advice that coaches who work with male competitive cyclists should be particularly aware of bone fractures and encourage their athletes to monitor bone mineral density over the course of their careers. It is also recommended that cyclists take the appropriate precautions to assess their present bone health. In order to protect the bone health of male cyclists, coaches and health care professional should also consider implementing a strength training program.

*Type: resistance training.*

*Duration: mean of 26.5 minutes/week (Standard deviation: 42.3 minutes).*

*Frequency: Not reported*

*Intensity: Not reported*

*Description: Cyclists completed a self-report questionnaire that included questions about their weekly hours of cycling training, weekly minutes of weight training, and years of competitive cycling experience.*

*Prior to the laboratory visit, participants completed a 1-day dietary recall, reflecting a typical day of their usual diet, to estimate their calcium intake.*

*16% of cyclists (aged < 50) had lumbar spine bone mineral density below the expected range for age. The number of minutes spent weight training weekly was found to have a strong positive correlation with bone mineral density of the lumbar spine, total hip, femoral neck, and femoral trochanter. A negative correlation was noted between age and the number of minutes spent weekly in weight training.  

Bivariate linear regression analyses showed that the number of weekly minutes spent on weight training was associated with higher bone mineral density at the lumbar spine (p = < 0.001), total hip (p = < 0.001), femoral neck (p = < 0.001), and femoral trochanter (p = < 0.001). Calcium intake was associated with greater bone mineral density at the lumbar spine (p = 0.06). Years of cycling experience was associated with lower femoral neck bone mineral density (p = 0.02).

Multivariable linear regression analysis was conducted to examine the relationship between age, calcium intake, weekly minutes of cycling and weight training, with bone mineral density at four sites: lumbar spine, total hip, femoral neck, and femoral trochanter. The analysis showed that weight training was the only variable significantly and positively associated with higher bone mineral density: lumbar spine (p = 0.007), total hip (p = < 0.001), femoral neck (p = < 0.001), and femoral trochanter (p = < 0.001). Specifically, as the number of weekly minutes spent on weight training increased, bone mineral density at all four sites increased.*

*40 male competitive amateur cyclists, between the ages of 31-69 years old, were contacted at the beginning of a road bicycle racing season.*

*The purpose of this cross-sectional study was to assess the dietary and exercise variables associated with bone mineral density in male competitive cyclists.*

*Bone mineral density at lumbar spine (L1-L4), total hip, femoral neck, and femoral trochanter was assessed by using dual X-ray absorptiometry.*

*In comparison to sedentary controls, road cyclists have reduced bone mineral density, especially at the pelvis, but similar levels at other sites, except for the head. Whole-body vibration training appears to improve hip bone mineral density and maintain spine bone mineral density. However, the duration of ten weeks may not be adequate to fully demonstrate the benefits of this training. Longer training periods, larger sample sizes, and a more controlled approach to cycling training regimes should all be part of further research.*

*Prioreschi et al. 2012, South Africa (34)*

*Whole Body Vibration Increases Hip Bone Mineral Density in Road cyclists*

*At baseline, pelvic bone mineral density was significantly lower in the intervention group (p = 0.020) and cyclist control group (p = 0.049) compared to the sedentary control group. Head bone mineral density was significantly greater in the intervention group (p = 0.008) and cyclist control group (p = 0.040) compared to the sedentary control group.*

*After the 10-week intervention period, hip bone mineral density in the intervention group increased significantly compared to the cyclist control group (p = 0.024). During the intervention period, cyclists in the control group lost significantly more spine bone mineral density (p = 0.020) compared to the intervention group (p = 0.590). However, this difference was no longer significant after adjusting for baseline spine bone mineral density measures.*

*Of the included participants, one cyclist reported back pain during the whole-body vibration. It remains unclear if this was due to a pre-existing condition or the intervention. As a result, this participant was excluded. Two more participants in the intervention group were excluded as they did not complete logbooks regularly. Compliance was not reported.*

*Type: whole-body vibration training.*

*Duration: 10 weeks, 15 minutes/session.*

*Frequency: 3 times/week*

*Intensity: 15 minutes consisting of 10 sets of 60s of whole-body vibration, and 30s of rest. 30 Hertz, and amplitude of 3 millimeters.*

*Description: Standing barefoot on vibration plates with slightly bent knees, holding firmly onto handlebars, placing most pressure on the heel.*

*Each week, participants completed logbooks detailing the minutes spent on the vibration plates, including frequency and amplitude. These logbooks were reviewed weekly to monitor compliance to the intervention protocol.*

*In total there was 36 participants aged 19 to 59: 18 trained to well-trained cyclists, and 18 sedentary controls.

Road cyclists was divided into two groups: intervention group (n = 11), and control group (n= 7). Road cyclists in the intervention group performed whole-body vibration training in addition to their normal cycling training, while the control group continued with their normal cycling training for 10 weeks.

The cyclists were closely matched in terms of age, sex, height, and body mass with a sedentary participant (n = 18).*

*The aim of this randomized controlled study was to compare bone mineral density between well-trained road cyclists and sedentary individuals, and to investigate the effects of a 10-week intervention of whole-body vibration training on bone mineral density of the cyclists.*

*Dual X-ray absorptiometry was used to assess the bone mineral density at baseline and after the 10-week intervention period.*

**Additional file 3.** (Continued)

Author, year and country Method/design/aim Study population Intervention Results Conclusion
